# Deep Learning Application to Clinical Decision Support System in Sleep Stage Classification

**DOI:** 10.3390/jpm12020136

**Published:** 2022-01-20

**Authors:** Dongyoung Kim, Jeonggun Lee, Yunhee Woo, Jaemin Jeong, Chulho Kim, Dong-Kyu Kim

**Affiliations:** 1 Department of Computer Engineering, Hallym University, Chuncheon 24252, Korea; kimdongyoung0218@hallym.ac.kr (D.K.); jeonggun.lee@hallym.ac.kr (J.L.); wyh@hallym.ac.kr (Y.W.); jaemin.jeong@hallym.ac.kr (J.J.); 2 Department of Neurology, Chuncheon Sacred Heart Hospital, Hallym University College of Medicine, Chuncheon 24252, Korea; gumdol52@hallym.or.kr; 3 Institute of New Frontier Research, Division of Big Data and Artificial Intelligence, Chuncheon Sacred Heart Hospital, Chuncheon 24252, Korea; 4 Department of Otorhinolaryngology-Head and Neck Surgery, Chuncheon Sacred Heart Hospital, Hallym University College of Medicine, Chuncheon 24252, Korea

**Keywords:** deep learning, sleep scoring, neural network, EEG, sleep staging

## Abstract

Recently, deep learning for automated sleep stage classification has been introduced with promising results. However, as many challenges impede their routine application, automatic sleep scoring algorithms are not widely used. Typically, polysomnography (PSG) uses multiple channels for higher accuracy; however, the disadvantages include a requirement for a patient to stay one or more nights in the lab wearing uncomfortable sensors and wires. To avoid the inconvenience caused by the multiple channels, we aimed to develop a deep learning model for use in clinical decision support systems (CDSSs) and combined convolutional neural networks and a transformer for the supervised learning of three classes of sleep stages only with single-channel EEG data (C4-M1). The data for training, validation, and test were derived from 1590, 341, and 343 polysomnography recordings, respectively. The developed model yielded an overall accuracy of 91.4%, comparable with that of human experts. Based on the severity of obstructive sleep apnea, the model’s accuracy was 94.3%, 91.9%, 91.9%, and 90.6% in normal, mild, moderate, and severe cases, respectively. Our deep learning model enables accurate and rapid delineation of three-class sleep staging and could be useful as a CDSS for application in real-world clinical practice.

## 1. Introduction

Sleep is an essential part of our daily lives, with multiple health problems arising from sleep disorders. Numerous studies have demonstrated that sleep disorders can cause or exacerbate severe major organ disorders, such as cardiovascular disease and neurocognitive deterioration [1,2,3]. Untreated sleep disorders are also a significant contributor to motor vehicle accidents [4,5]. Detecting these sleep disorders requires accurate interpretation of physiological signals. Currently, overnight polysomnography (PSG) is the “gold standard” for investigating sleep disorders, such as the evaluation of sleep stage, respiration, and limb movement [6]. However, PSG scoring is labor-intensive and is prone to variability in inter- and intra-rater reliability [7,8,9,10,11]. Currently, manual sleep scoring is the gold standard, requiring trained sleep technicians to apply visual pattern recognition to the signals. Under ideal circumstances, interrater reliability among scores approaches 0.90, and direct percent agreement approaches 80%, whereas, in clinical settings, these agreement metrics are typically lower, even with quality oversight [9,12,13]. Therefore, attempts to automate this process have been extensively explored since 2000 [14].

Recently, several studies on deep neural networks using labeled large datasets have matched the performance of medical experts in complex medical pattern recognition tasks [15,16,17]. In the field of sleep medicine, some studies also reported a higher accuracy of sleep stage evaluation using PSG data and suggested the feasibility of a deep learning algorithm for sleep stage scoring as a clinical decision support system (CDSS) [18,19]. The use of an automated CDSS is one way to establish a reliable diagnosis with easy access and convenience [20,21]. Additionally, confidence in the CDSS system should be established by traceability [22]. To date, almost all studies have shown remarkable accuracy using multi-channel EEG for sleep stage scoring [14,23]. However, in [24], the authors described that PSG using multiple channels has disadvantages requiring the patient to stay one or more nights in the lab wearing uncomfortable sensors and wires. In [25,26], it was reported that numerous leads placed on the patient are necessarily involved with the discomfort due to restricted movement. In addition to PSG, in [27], the authors suggested single EEG channel approach for developing brain–computer interface (BCI) systems. In general, BCI uses a large number of multiple EEG channels (more than that of PSG). The authors mentioned that it is inconvenient and uncomfortable to place multiple electrodes on the scalp.

Considering the complexity of the sleep process itself and of sleep disorders, the information of many channels is still essential for rigorous scoring; however, single-channel-based scoring is indeed a promising approach [28,29,30,31,32,33,34,35] because it could be easily used for monitoring patients in intensive care units or nursing hospitals. Additionally, sleep stage scoring based on multiple channels has a complex PSG setup owing to the deployment of multiple sensors on the patient’s head and body. The significant number of sensors required makes it difficult to develop portable or mobile PSG test devices. In [36], the authors used only a single-channel EEG and performed real-time sleep stage classification. The goal of the paper was to simplify and automate PSG on a smartphone for automatic and real-time interventions that can potentially be used in future human–computer interaction (HCI) applications. In our approach, the aim of real-time processing is for providing real-time status of patients to “the person such as medical doctors or nurses who needs such information for real-time patient care” or to “the medical devices which are attached to a patient body for assist the patient in real-time”. Thus, if CDSS gives information regarding real-time sleep staging, we could use it to elucidate the ventilator mode or to monitor real-time sleep status for patients who live in nursing hospitals.

For these reasons, we aimed to develop a novel deep learning algorithm for sleep stage scoring (three classes) using a single EEG channel because multiple channels are very inconvenient to the patients in real-world practice. Moreover, future epochs were not used to classify the current epoch. Only current and previous epochs were used to predict the sleep stage of a current epoch. Consequently, our deep learning approach can be used in real-time classification, in which future information cannot be used for classification of the current epoch.

## 2. Materials and Methods

This study was approved by the Institutional Review Board of Chuncheon Sacred Heart Hospital, Hallym University College of Medicine (Chuncheon, Republic of Korea: No. 2021-03-005). Written informed consent was waived because the study used data from a de-identified database. To protect patients’ confidentiality, only the manager of the database could access both identified and de-identified codes. Thus, we obtained the anonymous dataset used in the study from the manager.

### 2.1. Preparation of Study Dataset

We retrieved PSG data from our sleep center involving patients with and without sleep-disordered breathing. At our sleep center, standard overnight PSG was performed preoperatively for all patients using a computerized polysomnographic device (Nox-A1, Nox Medical Inc. Reykjavik, Iceland). PSG records various bio-signal data, including combinations of electroencephalogram (EEG), electrooculogram (EOG), electromyogram (EMG), electrocardiography (ECG), and respiratory signals (chest belt, abdomen belt, oximetry, and airflow). The distribution of the PSG dataset was created indirectly by a mixture of diagnostic, split night, and titration protocols. Additionally, the PSG dataset was labeled with event annotations by certified sleep technologists according to the guidelines of the American Academy of Sleep Medicine (AASM, version 2.6) [6].

### 2.2. Data Curation and Preprocessing

**Figure 1:**
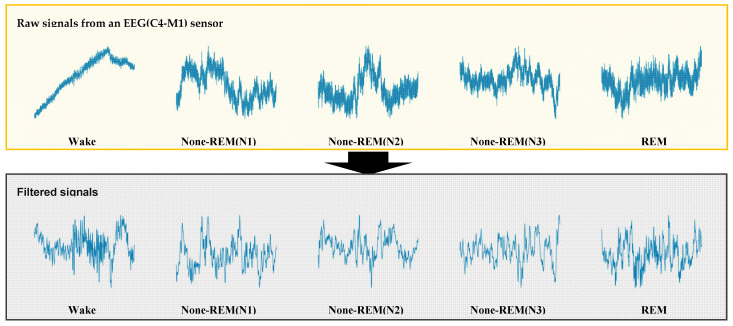
Representative raw data sample from each sleep stage. Bandpass filtering was applied to raw polysomnographic data to reduce impact of noise and for artifact reduction.

**Figure 2:**
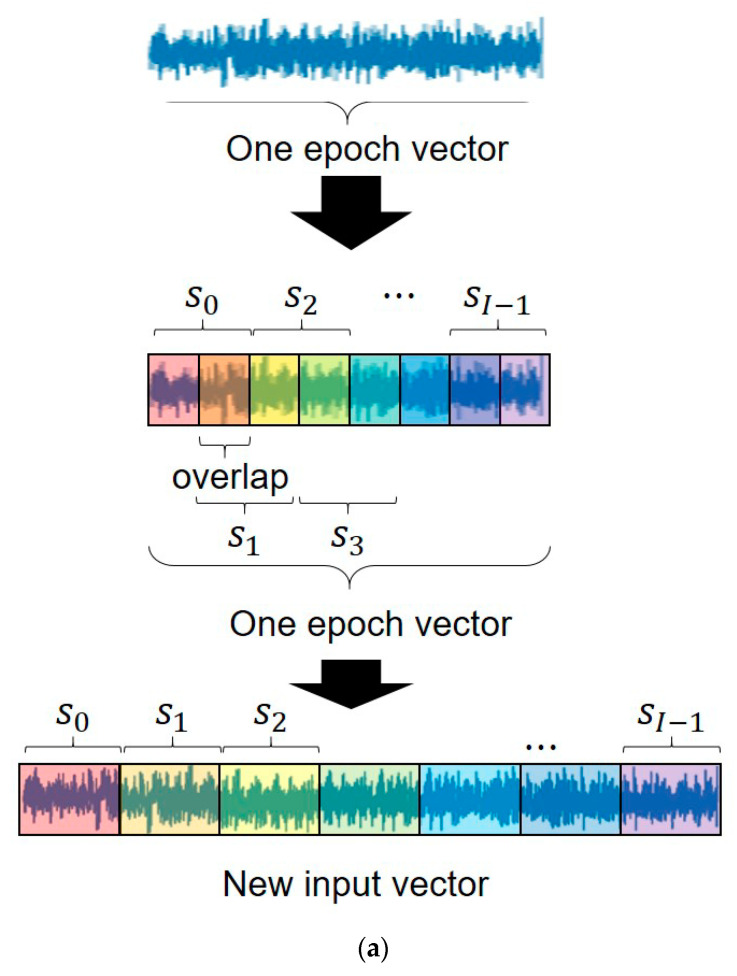

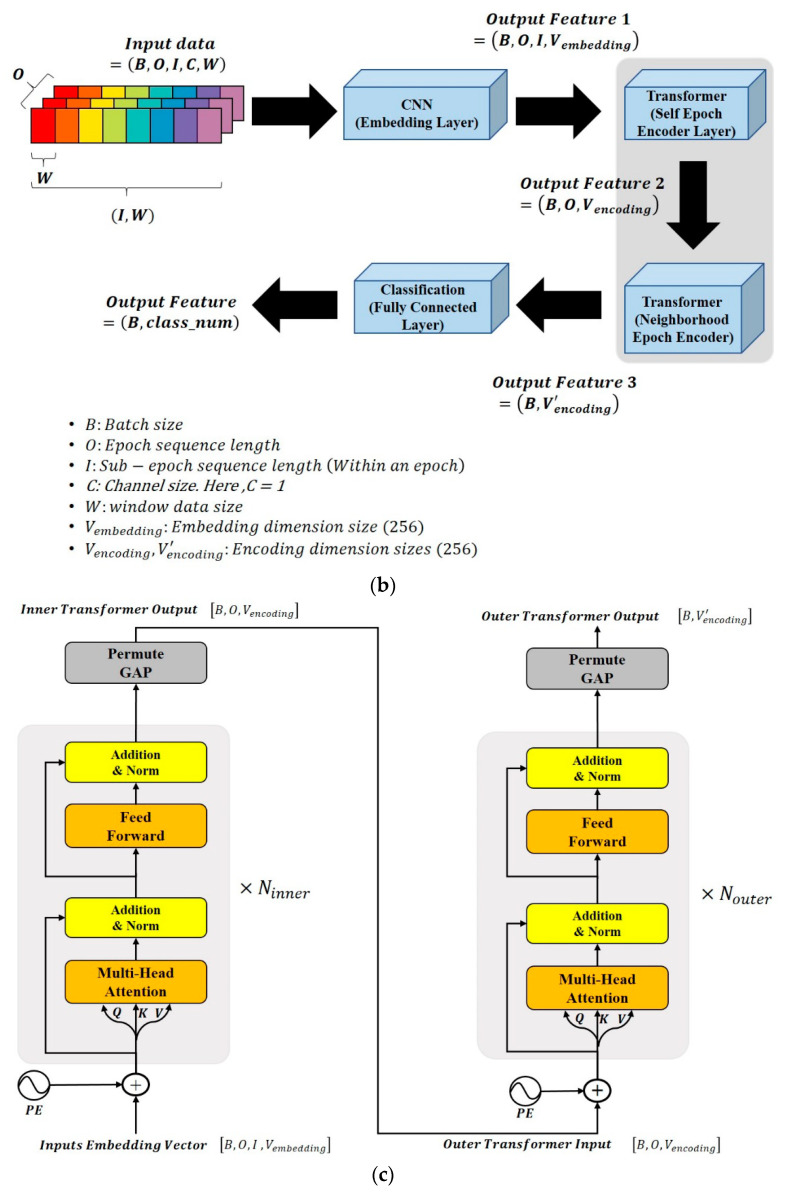
Simplified deep learning model architecture for automated polysomnography analysis. (**a**) Sequential information must be created in a single epoch to develop input data from preprocessed data. At this time, in the case of 𝑊𝑖𝑛𝑑𝑜𝑤 > 𝑆𝑡𝑟𝑖𝑑𝑒, overlap as much as the difference occurs. (**b**) Overall end-to-end architecture of our deep learning model based on transformers with a CNN. (**c**) Architecture of inner/outer transformer models with independent sets of weight parameters.

For sleep staging, EEG signals were scored in non-overlapping 30 s epochs according to the AASM standards as one of five stages: wake (W), rapid eye movement (REM, R), non-REM stage 1 (N1), non-REM stage 2 (N2), and non-REM stage 3 (N3). Therefore, sleep staging was formulated as a 5-class classification problem. EEG data in PSG consisted of signals from six channels (i.e., F3, F4, C3, C4, O1, and O2) and each referenced to the contralateral mastoid. In this study, we targeted a classification problem for the three classes: W, N (N1, N2, N3), and R, using a single central (C4) EEG channel. Then, a deep learning model was developed with labeled PSG data by employing supervised learning to classify the three stages. These classes have ~0.37 million, 1 million, and 0.23 million 30 s epochs from our dataset for W, N (N1–N3), and R, respectively. We subsequently used raw numerical waveform data in the dataset as inputs for our models. The raw PSG signals were also preprocessed to train a deep learning model (Figure 1). We used preprocessing methods with a MinMax scaler and a bandpass filter for normalizing signal values and for reducing noise/artifact of signals. During the preprocessing procedure, we obtained 0.5–35 Hz signal information using the band-pass filter, and we added the MinMax scaler to normalize the descriptor values. To further exploit the temporal relations within the single epoch data, the sub-epoch data samples ($s_{0},s_{1},\ldots ,\;s_{I-1}$) were generated by sequentially moving the preprocessed input data of the window size (W) as much as the stride size in a single epoch (Figure 2a). The number of sub-epochs can be described using the following equation (e.g., for sample rate: 200/s, epoch size: 30 s, window size (*Window*): 800, and stride size (*Stride*): 400; 14 sub-epochs are extracted from a single epoch).

(1) $$I=\frac{\left(sample\;rate\left(200/s\right)\times epoch\;size\left(30\;s\right)-Window\left(800\right)\right)}{Stride\left(400\right)}+1=14$$

Finally, the preprocessed PSG signal data were provided directly as input to the deep neural network in per-epoch units. To develop a deep learning model, we split our datasets into training, validation, and test datasets using 70/15/15 percentage splits of patients. The summary of the partitioned training, validation, and test datasets is presented in Table 1.

### 2.3. Deep Learning Model Architecture

Our deep learning model was implemented using the Python programming language. Recently, attention on transformer-based deep learning models have been proposed and investigated. In this study, our deep learning model consisted of a convolutional neural network (CNN), an inner transformer, an outer transformer, and a classifier. The CNN was created using five convolutional layers. The deep neural network was built by cascading a CNN, two consecutive transformer structures, and a classifier trained with the preprocessed input signals generated from the raw data (Figure 2b,c). Our training process consisted of two steps. The first step was the inner transformer model with a CNN to train each epoch. The CNN was used as an embedding layer for extracting key features of input sub-epoch data and for generating embedding vectors (vector dimension is $V_{embedding}$) corresponding to sub-epoch data. With the patches of the embedding vectors extracted from the sub-epoch, the inner transformer produced the encoded features (feature dimension is $V_{encoding}$) to describe the class of a single 30 s epoch data. The inner transformer was designed to capitalize on the temporal relationships between the embedding vectors corresponding to the sub-epochs in a given epoch and thereby produce feature maps that determine the class of the given epoch.

The second step was a fine-tuning process with a sequence of multiple consecutive epochs. The outer transformer model used the encoded features obtained from the inner transformer. The outer transformer fine-tuned the feature encoding vectors (feature dimension is $V_{encoding}^{\prime }$) to better classify a target epoch by further considering the temporal relations from previous neighboring epochs. In general, previous studies used neighboring epochs located before and after the current epoch. However, in this study, the sleep stage classification model solely used current and previous epochs for current epoch classification without considering future epochs for the application of real-time classification. Finally, a classifier determined the classes (class_num) from the feature-encoding vectors through the fully connected layers.

During the training process, the Adam optimizer was used with a learning rate of 0.0001 and a decay rate dependent on the cosine annealing scheduler. We used cross-entropy as a loss function, and batch normalization was applied after each convolutional layer.

### 2.4. Deep Learning Model Training and Validation

**Figure 3:**
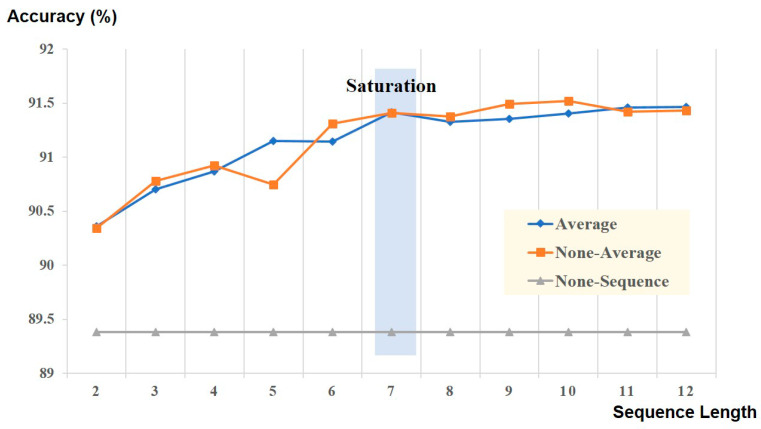
Performance of deep neural network model based on sequence length. The learning curve begins to plateau when more than 7 sequential epochs are used.

Extraction of the feature embedding vectors and training the inner transformer required ~4.6 h (for 51 iterations), and model fine-tuning required ~3.8 h (for 15 iterations) on an Nvidia RTX 3090 GPU. We observed that improvement in accuracy was saturated when more than seven consecutive epochs (the current epoch and six previous consecutive epochs) were utilized for the outer transformer in both the average and non-average models (Figure 3). An accuracy of 91.4% was achieved with seven consecutive epochs, and a 2.02% improvement was obtained when compared to the case of using a single epoch (89.38%).

### 2.5. Deep Learning Model Testing and Evaluation

In this study, the PSG sets used for training, validation, and testing were kept constant. There was no overlap between test and training sets. Model performance was evaluated on the test sets with recall, precision, F1 score, and weighted/unweighted accuracy to assess the effect of sleep stage class imbalances in this dataset. The weighted accuracy was calculated as the average of the per-class accuracy. Transition epoch F1-scores were calculated because scoring agreement is known to degrade during the transition from one stage of sleep to another. Transition stages accounted for ~0.5% of the data, but were nevertheless evaluated, as they potentially convey physiologically relevant information.

## 3. Results

**Figure 4:**
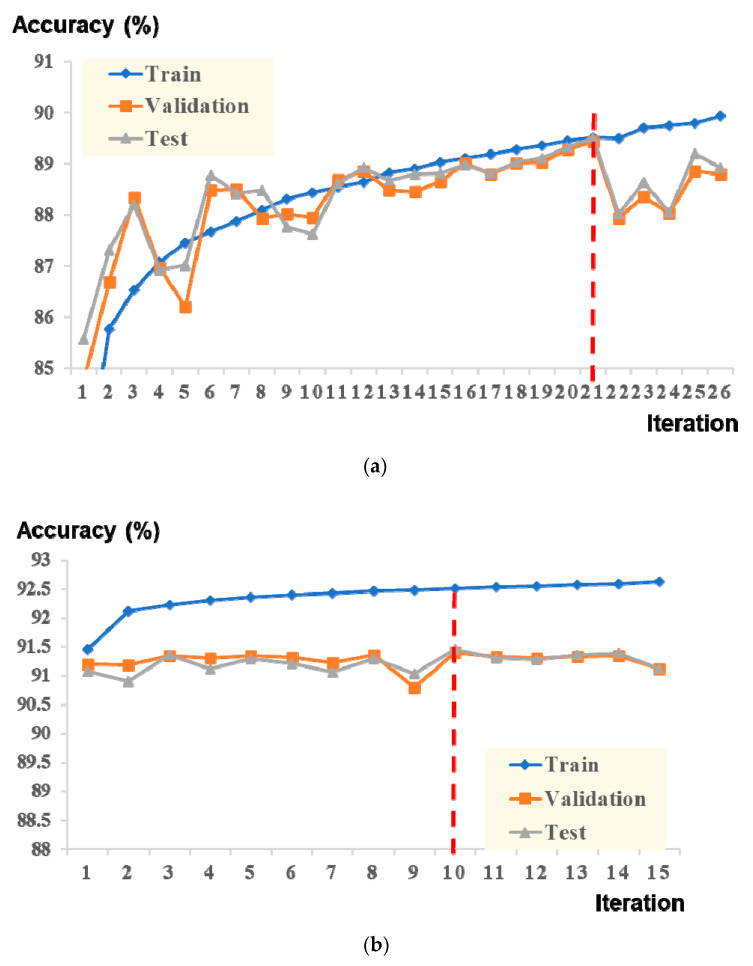
Evaluation of training times for best training accuracy. One iteration means that all the data samples in the entire training set have been used to train a deep learning model. (**a**) Training/validation/test accuracy trend for a single-epoch model. (**b**) Training/validation/test accuracy trend for a multi-epoch model.

Our data consisted of 2274 clinical PSGs performed at the Hallym University Sleep Laboratory, divided into training (n = 1590), validation (n = 341), and test (n = 343) datasets. In addition, each dataset comprised an equal distribution of OSA severities (Table 2). Preprocessing methods were applied to raw input signal data before being used for training. It is noteworthy that no significant improvement in accuracy or F1 scores was found using preprocessing, such as normalization or bandpass filtering; however, through the preprocessing, the learning speed was slightly improved. Consequently, preprocessing was used in the final pipeline. In the present study, we developed a novel deep learning model for automated three-class sleep staging and a deep learning model consisting of CNN and two-stage transformer architectures. When the feature embedding on the convolutional layer and inner transformer was trained, the highest accuracy was achieved at the 21st iteration (Figure 4a). The best-trained weight parameters of the CNN and inner transformer were then frozen. Subsequently, the weight parameters of the outer transformer and classification layer were trained. The highest accuracy was obtained at the 10th iteration (Figure 4b).

**Figure 5:**
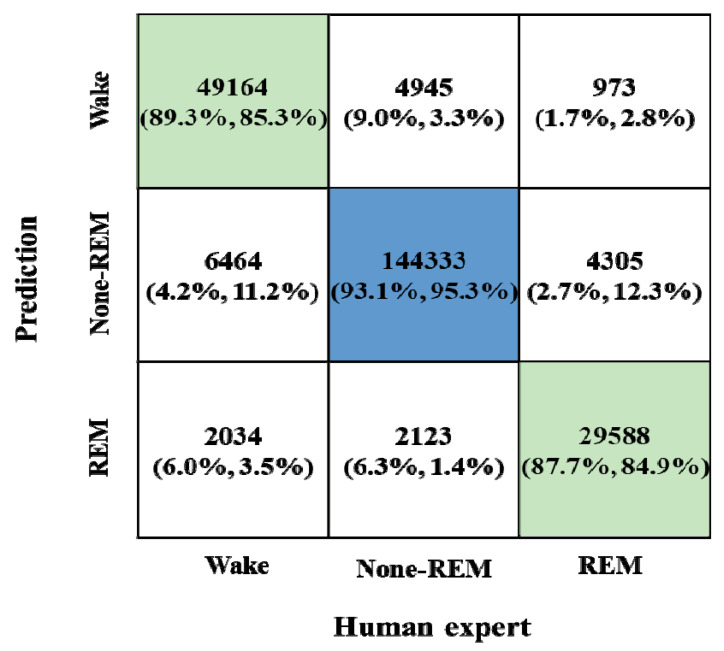
Classification performance of the deep learning model for sleep stage scoring. Confusion matrix showing numbers of samples classified correctly or incorrectly as a percentage: precision and recall values for each case.

For three-class sleep staging, our deep learning model based on the two-stage transformers with CNN achieved an overall accuracy of 91.45%, which compares favorably to human expert performance (Table 3). Additionally, this model achieved a macro F1-score of 0.89, Cohen’s unweighted kappa of 0.84, and balanced accuracy of 0.8849. A confusion matrix was generated for the model performance against all tested epochs (Figure 5). When considering all epochs, the model scored Wake, NREM, and REM stages as 89%, 93%, and 88% for precision and 85%, 95%, and 85% for recall, respectively. To figure out the impact of the OSA severity on the accuracy performance of the developed model, we used different testing datasets according to the OSA severity. In this process, we first developed a deep neural network by training all the training datasets without considering the levels of OSA severity. Then, the model performance was evaluated with the testing datasets of different OSA severity levels to evaluate recall, precision, F1 score, and weighted/unweighted accuracy. As shown in Table 4, the accuracy for the test dataset of the OSA severity was 94.18% in normal, 93.82% in mild, 91.60% in moderate, and 90.39% in severe cases.

**Figure 6:**
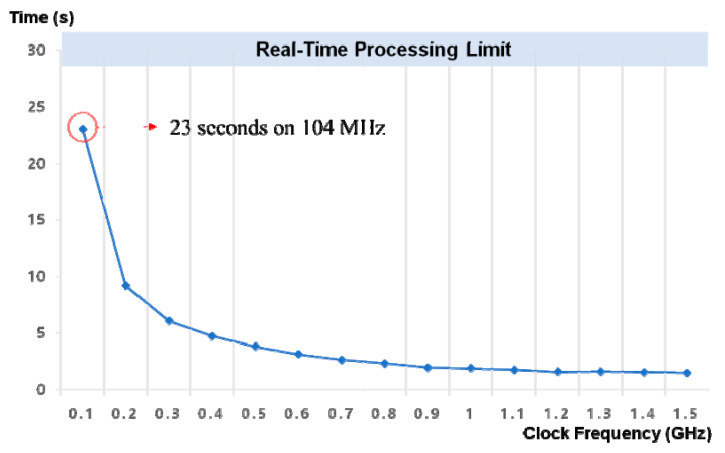
Performance of the real-time processing for sleep stage classification. The inference speed with O=7, I=14, W=800 according to the clock frequency of the CPU core.

To confirm the feasibility of the CDSS application, we investigated the performance of real-time interpretation for three-class sleep staging. Figure 6 demonstrates the inference time for classifying a target 30 s epoch sample according to the core clock frequency. In this experiment, we disabled the use of embedded GPUs to evaluate the inference performance using only CPU cores. We observed that the inference could be performed within 30 s, even at a 104 MHz core clock frequency. This means that our deep learning model can be successfully utilized for real-time inferences, even with CPU cores operating at low clock frequencies. The inference time approached 1.48 s as the core clock frequency was increased to 1.5 GHz.

Table 5 shows a comparison between our work and other recent state-of-the-art methods in terms of overall accuracy. For fair comparisons, we used the same dataset and same training, validation, and test splitting policy mentioned in Table 1 to evaluate model accuracies for the existing works and our work. Note that our model achieved the best results.

Additionally, we tested all the possible individual channels, including C3, C4, E1, E2, F3, F4, O1, and O2 to evaluate the accuracies of sleep staging for the cases of utilizing only a single channel. The detailed performance comparison was presented in Table 6. As shown in the table, the deep learning model using the C4 channel showed the best performance in both single-epoch and multi-epoch models. Thus, the C4 channel was selected for use in our single channel-based CDSS. Next, to investigate the performance impact of multiple channel combinations, we evaluated deep learning model performance according to various channel combinations and the obtained results are presented in Table 7. As shown in Table 7, the C4 channel-based single-channel model showed comparable accuracy performance (91.45%) when compared to the performance of the multi-channel models.

Finally, to show the extensibility of the proposed model, we used another well-known SHHS public dataset to train and test the model. Table 8 shows the detailed training/validation/test partitioning information for training the SHHS (Sleep Heart Health Study) public dataset. The SHHS dataset has the same partitioning strategy used in our own data partitioning (as presented in Table 1). Table 9 shows the results for the performance of the model using the SHHS test dataset. Although the model uses a single-channel EEG, meaningful performance accuracy could be demonstrated. Particularly noteworthy is that 92.26% and 91.82% performance accuracy was achieved for REM class prediction with only single-channel EEG data from C4-A1 and C3-A2, respectively.

## 4. Discussion

In the present study, we developed a deep learning model for sleep stage scoring and demonstrated its utility as a CDSS. Our deep learning model showed strong agreement with expert human scorers. The proposed model works based on information obtained from only a single-channel EEG sensor and operates in real-time by considering only current and previous epoch data. We also confirmed that preprocessing raw signal data for noise and artifact reduction did not significantly affect the sleep staging results. Therefore, although our deep learning model presented a three-class sleep stage, it could also be applied for CDSS in the field of clinical medicine, such as in conditions requiring adjustment of the ventilator mode in chronic respiratory patients under continuous monitoring.

To date, various studies have been proposed to achieve very favorable results in terms of accuracy [29,32,34,37]. Nevertheless, automatic sleep scoring algorithms have not yet been implemented in sleep centers worldwide, although clinical sleep scoring involves a tedious visual review of overnight PSG by expert human scorers. For these reasons, we aimed to develop a deep learning model suitable for real-world application. CDSS, which includes a variety of tools and interventions computerized, as well as non-computerized, could provide aid for clinical decision making [38]. High-quality CDSS can support clinical decision-making in daily clinical practice [39]. Therefore, we focused on the development of a deep learning model that can be utilized for CDSS.

In addition, several previous studies reported a deep learning model based on single-channel EEG signals because of the requirement for a reliable solution with few channels [29,32,34,37]. Additionally, some studies showed the data for sleep staging using only the EOG channel without EEG [40,41,42]. For single-channel EEG signals, our deep learning model showed an accuracy rate of 91.45% and a balance accuracy of 88.49%. We believe this accuracy belongs to the acceptable range because higher performance could be considered as overfitting on the dataset [23]. 

Moreover, we confirmed the performance of real-time processing for sleep stage classification using a mobile edge device. Interestingly, we found that the CNN with transformer model provided the inference within 30 s even at 104 MHz core clock frequency; the inference time also decreased to 1.48 s as the core clock frequency increased to 1.5 GHz. Owing to the fact that one epoch of PSG is 30 s, we believe that our deep learning model is feasible for application in CDSS. While most previous studies on sleep staging algorithms used CNN with recurrent neural network (RNN) as the model architecture, our deep learning model used CNN with the transformer. Generally, an RNN model works in a sequential manner, whereas transformers can process sequence input data simultaneously using matrix multiplication. One of our main goals was to apply the developed deep learning model to the CDSS; as a result, it should work in real time in real-world applications. Additionally, we investigated the optimal epoch sequence for training and discovered that seven epochs (six previous epochs and the current epoch) were the optimal sequence for the architecture of the CNN with the transformer.

In conclusion, we demonstrated the use of a CNN with transformer models for the automatic detection of sleep stage events using a single-channel EEG signal. The transformer consists of an inner transformer and an outer transformer classifier. Additionally, the proposed algorithm can optimize the performance in real-world clinical settings; therefore, it can be used as a CDSS. In the future, the model could be employed for the detection of other sleep-related abnormalities.

## Figures and Tables

**Table 1:** Summary of datasets.

Dataset Type (No. of Patients)	Wake	Non-REM	REM
Training dataset (1590)	262,511 (23%)	702,824 (62%)	163,277 (15%)
Validation dataset (341)	53,644 (23%)	147,607 (63%)	34,761 (14%)
Testing dataset (343)	57,662 (23%)	151,401 (62%)	34,866 (14%)

**Table 2:** Profile of datasets based on the severity of obstructive sleep apnea.

Dataset	Severity	Wake	Non-REM	REM
Training (1590)	Normal (109)	19,062 (23%)	50,660 (61%)	13,323 (16%)
	Mild (229)	34,908 (21%)	104,518 (62%)	28,373 (17%)
	Moderate (336)	57,794 (23%)	149,596 (61%)	38,747 (16%)
	Severe (916)	150,747 (24%)	398,050 (63%)	82,834 (13%)
Validation (341)	Normal (23)	3066 (18%)	11,163 (64%)	3145 (18%)
	Mild (49)	7341 (21%)	22,227 (63%)	5556 (16%)
	Moderate (72)	11,995 (24%)	31,829 (62%)	7200 (14%)
	Severe (197)	31,242 (24%)	82,388 (62%)	18,860 (14%)
Testing (343)	Normal (24)	3260 (18%)	11,952 (65%)	3200 (17%)
	Mild (50)	8367 (23%)	22,677 (62%)	5326 (15%)
	Moderate (72)	12,443 (23%)	32,361(61%)	8576 (16%)
	Severe (197)	33,592 (25%)	84,411 (62%)	17,764 (13%)

**Table 3:** Deep learning model performance for three-class sleep scoring.

	Wake	Non-REM	REM
Recall	0.85	0.95	0.85
Precision	0.89	0.93	0.88
F1 score	0.87	0.94	0.86
Cohen’s Kappa		0.84	
Macro F1-score		0.89	
Weighted accuracy		88.49%	
Accuracy		91.45%	

**Table 4:** Performance of deep learning model based on the severity of obstructive sleep apnea.

	Cohen’s Kappa	Macro-F1-Score	Weighted Accuracy	Accuracy
Normal	0.89	0.92	92.35%	94.18%
Mild	0.88	0.92	92.10%	93.82%
Moderate	0.85	0.89	89.55%	91.60%
Severe	0.82	0.87	87.86%	90.39%

**Table 5:** Comparison of performance between previous studies and the present study.

	Model	Method	Class-Wise Recall			Class-Wise Precision			Overall Metrics
			Wake	NREM	REM	Wake	NREM	REM	Accuracy
Single-Epoch	DeepSleepNet [34]	CNN	0.76	0.93	0.74	0.90	0.89	0.68	86.06
	AttnSleep [35]	CNN	0.80	0.94	0.78	0.90	0.91	0.78	88.71
	The present work	Transformer	0.83	0.95	0.79	0.89	0.91	0.81	89.50
Multi-Epoch	DeepSleepNet [34]	CNN + RNN	0.84	0.95	0.78	0.87	0.92	0.86	89.88
	AttnSleep [35]	CNN + RNN	0.82	0.96	0.85	0.91	0.92	0.85	90.93
	The present work -1	Transformer + RNN	0.85	0.95	0.86	0.89	0.93	0.86	91.38
	The present work -2	Inner + Outer Transformer	0.85	0.95	0.85	0.89	0.93	0.88	91.45

**Table 6:** Accuracy performance of a single channel-based deep learning model according to the channel types (EEG/EOG) and positions.

	Channel		Class-Wise Recall			Class-Wise Precision			Overall Metrics
			Wake	NREM	REM	Wake	NREM	REM	Accuracy
Single-Epoch	EEG	C3-M2	0.81	0.95	0.75	0.90	0.90	0.81	88.97
		C4-M1	0.83	0.95	0.79	0.89	0.91	0.81	89.50
		F3-M2	0.82	0.95	0.80	0.90	0.91	0.81	89.44
		F4-M1	0.80	0.95	0.83	0.91	0.91	0.79	89.40
		O1-M2	0.78	0.92	0.79	0.89	0.90	0.69	86.74
		O2-M1	0.78	0.93	0.78	0.90	0.90	0.73	87.46
	EOG	E1-M2	0.81	0.95	0.81	0.89	0.91	0.82	89.32
		E2-M1	0.81	0.93	0.84	0.89	0.92	0.78	89.07
Multi-Epoch	EEG	C3-M2	0.85	0.95	0.83	0.89	0.93	0.87	91.10
		C4-M1	0.85	0.95	0.85	0.89	0.93	0.88	91.45
		F3-M2	0.86	0.95	0.84	0.88	0.93	0.88	91.21
		F4-M1	0.87	0.94	0.84	0.87	0.94	0.88	91.18
		O1-M2	0.83	0.94	0.82	0.89	0.92	0.82	89.79
		O2-M1	0.83	0.95	0.82	0.88	0.92	0.85	90.17
	EOG	E1-M2	0.83	0.96	0.86	0.90	0.93	0.88	91.27
		E2-M1	0.83	0.96	0.86	0.90	0.93	0.87	91.23

**Table 7:** Deep learning model performance according to channel combinations.

Channel		Class-Wise Recall			Class-Wise Precision			Overall Metrics
		Wake	NREM	REM	Wake	NREM	REM	Accuracy
C4-M1		0.85	0.95	0.85	0.89	0.93	0.88	91.45
C4 + EMG		0.85	0.95	0.89	0.90	0.94	0.86	91.70
C4 + E2(EOG)		0.86	0.95	0.89	0.89	0.94	0.89	92.16
C4 + EMG + E2(EOG)		0.87	0.95	0.90	0.89	0.95	0.88	92.27
C4 + F4 + O2 + EMG + E2(EOG)		0.88	0.95	0.90	0.88	0.95	0.89	92.41
Multi-EEG	2 EEG	0.86	0.95	0.88	0.90	0.94	0.87	92.02
	3 EEG	0.86	0.95	0.89	0.90	0.94	0.88	92.24
	4 EEG	0.84	0.96	0.87	0.90	0.93	0.87	91.76
	5 EEG	0.87	0.95	0.89	0.89	0.94	0.89	92.47
	6 EEG	0.85	0.96	0.88	0.91	0.94	0.89	92.33

**Table 8:** Summary of the SHHS dataset.

Dataset Type (No. of Patients)	Wake	Non-REM	REM
Training dataset (3884)	1,134,126 (29%)	2,247,931 (57%)	548,083 (14%)
Validation dataset (832)	243,558 (29%)	482,490 (57%)	116,685 (14%)
Testing dataset (834)	242,631 (29%)	481,646 (57%)	119,010 (14%)

**Table 9:** Performance accuracy of a single-channel EEG-based deep learning model based on a public SHHS dataset.

	Channel		Class-Wise Recall			Class-Wise Precision			Overall Metrics
			Wake	NREM	REM	Wake	NREM	REM	Accuracy
Single-Epoch	EEG	C4-A1	0.821	0.961	0.653	0.958	0.856	0.817	87.69%
		C3-A2	0.822	0.939	0.766	0.943	0.879	0.777	88.08%
Multi-Epoch	EEG	C4-A1	0.891	0.960	0.835	0.951	0.920	0.879	92.26%
		C3-A2	0.895	0.943	0.864	0.930	0.928	0.856	91.82%

## Data Availability

The authors confirm that the data supporting the findings of this study are available within the article.
